# PreBP: an interpretable, optimized ensemble framework using routine complete blood count for rapid pathogen identification in bacterial pneumonia

**DOI:** 10.3389/fbinf.2025.1769816

**Published:** 2026-01-14

**Authors:** Xiaoxi Hao, Dingjian Liang, Yimin Shen, Cuimin Sun, Wei Lan

**Affiliations:** 1 School of Computer, Electronics and Information, Guangxi University, Nanning, China; 2 Department of Radiation Oncology, Minzu Hospital of Guangxi Zhuang Autonomous Region, Affiliated Minzu Hospital of Guangxi Medical University, Nanning, Guangxi, China; 3 Guangxi Colleges and Universities Key Laboratory of Multimedia Communications and Information Processing, Nanning, China

**Keywords:** bacterial pneumonia, complete blood count, ensemble learning, interpretable machine learning, pathogen identification, SHapley additive explanations

## Abstract

**Introduction:**

Bacterial pneumonia remains a major global health challenge, and early pathogen identification is important for timely and targeted treatment. However, conventional microbiological diagnostics such as sputum or blood culture are labor-intensive and time-consuming.

**Methods:**

We propose an interpretable ensemble learning framework (PreBP) for rapid pathogen identification using routinely available complete blood count (CBC) parameters. We analyzed 1,334 CBC samples from patients with culture-confirmed bacterial pneumonia caused by four major pathogens: *Pseudomonas aeruginosa*, *Escherichia coli*, *Staphylococcus aureus*, and *Streptococcus* pneumoniae. Pathogen labels were determined based on clinical culture results. Five machine learning models (extreme gradient boosting (XGBoost), multilayer perceptron neural network (MLPNN), adaptive boosting (AdaBoost), random forest (RF), and extremely randomized trees (ExtraTrees)) were trained as comparators, and PreBP was developed with metaheuristic-optimized hyperparameters. Key CBC biomarkers were refined using a dual-phase feature selection strategy combining Lasso and Boruta. To enhance transparency, SHapley additive explanations (SHAP) were applied to provide both global biomarker importance and local, case-level explanations.

**Results:**

PreBP achieved the best overall performance, with an AUC of 0.920, precision of 87.1%, and accuracy and sensitivity of 86.7%.

**Discussion:**

Because the framework relies on routine CBC measurements, it can generate interpretable predictions once CBC results are available, which may provide supplementary evidence for earlier pathogen-oriented clinical decision-making alongside culture-dependent workflows. Overall, PreBP offers an interpretable and computational approach for pathogen identification in bacterial pneumonia based on routine laboratory data.

## Graphical Abstract

**Figure:**
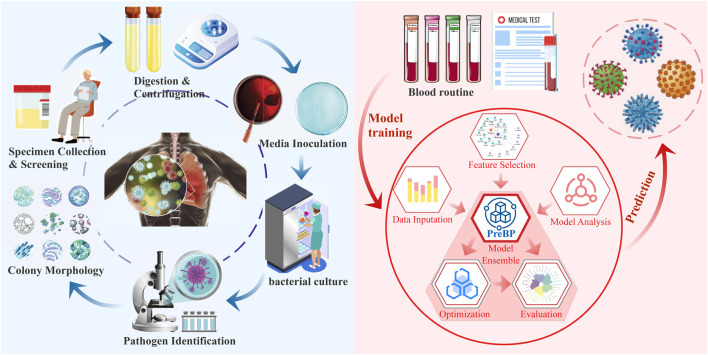

## 1 Introduction

Bacterial pneumonia remains a common cause of morbidity and mortality worldwide, highlighting the importance of timely and accurate pathogen identification. Notably, pneumonia-related morbidity and mortality disproportionately affect people 65 years of age and older (Castrodad-Rodriguez et al., 2021). According to a 2019 World Health Organization (WHO) report, 929,000 deaths were attributed to antimicrobial resistance (AMR) in six priority bacterial infections (Antimicrobial Resistance, 2022). Antimicrobial susceptibility testing (AST) and pathogen identification rely on blood culture, which takes 24–72 h to produce findings (Lamy et al., 2016). For life-threatening illnesses like septic shock, the treatment window is frequently less than 6 hours (Evans et al., 2021). Every year, some 4.1 million cases of empirical broad-spectrum antibiotic use are caused by this diagnostic delay (Cassini et al., 2019), which exacerbates the AMR epidemic.

**FIGURE 1:**
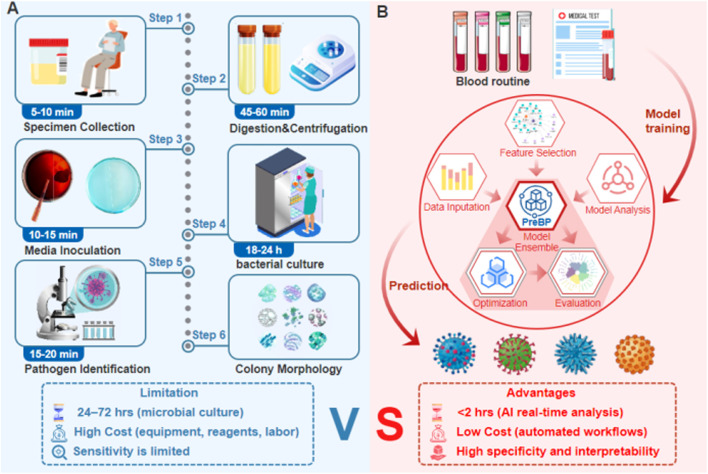
Integration of sputum culture diagnostics and the AI-driven pathogen prediction workflow **(A)** Qualified sputum samples are collected, processed through digestion, inoculated onto culture media, and incubated. Pathogen identification (via colony characterization) and antimicrobial susceptibility testing are performed to determine resistance profiles, guiding precision antimicrobial therapy in clinical practice. **(B)** Complete blood count (CBC) data (e.g., hematologic parameters) from patients infected with four bacterial types are used to train a model. By integrating metaheuristic optimization and interpretable AI techniques, a pathogen identification system is constructed, enabling rapid prediction of infection sources.

Current diagnostic techniques for pneumonia, such as sputum culture, molecular diagnostics (Figure 1A), and imaging analysis (Nordmann and Poirel, 2014), face significant limitations. Prolonged culture times result in 37% of patients receiving inappropriate antibiotics before AST results are available (Van Heuverswyn et al., 2023); Pretreatment with antibiotics reduces blood culture sensitivity to 42%–58% (Ruiz-Gaviria et al., 2023); Molecular assays achieve less than 63% specificity in distinguishing polymicrobial infections (Castrodad-Rodriguez et al., 2021). While biomarkers such as procalcitonin (PCT) assist differentiate bacterial from viral infections (Liang et al., 2023), their value in identifying specific pathogens is limited. Nanopore sequencing reduces pathogen identification time to 8 h but is prohibitively expensive (>US$200/test) (Guo et al., 2023), whereas AI models achieve high accuracy (AUC = 0.91) in pneumonia detection yet struggle with overlapping radiomic features among pathogens (Xu, 2023; Rognvaldsson et al., 2023).

To improve pneumonia diagnostics, machine learning (ML)-driven multimodal data fusion strategies have emerged as transformative tools. Studies integrating clinical data with machine learning techniques, such as XGBoost models and host immune response signatures, have demonstrated superior predictive performance (Li et al., 2024; Alzoubi and Khanfar, 2021). For example, an elevated neutrophil-to-lymphocyte ratio (NLR) in *Staphylococcus aureus* infections and increased platelet distribution width (PDW) in *Pseudomonas aeruginosa* bacteraemia highlight the utility of host immune fingerprints (HIFs) in pathogen discrimination (Sathe et al., 2023; She et al., 2023).

Moreover, predictive models leveraging peripheral blood parameters have shown promise in related contexts. A COVID-19 severity prediction model developed using peripheral blood parameters and ML algorithms achieves high accuracy (Aktar et al., 2021; She et al., 2023), and Oscar Garnica et al. developed models using Support Vector Machine, Random Forest, and K-Nearest Neighbors algorithms based on hospital electronic health records to predict bacteraemia, providing an efficient tool for personalized antimicrobial treatment decision-making and healthcare resource optimization (Garnica et al., 2021). Existing efforts have primarily focused on binary classification, highlighting the need for multiclass frameworks for clinical translation.

Despite progress, three major challenges remain: extracting discriminative immune biomarkers from high-dimensional heterogeneous data, developing precise multiclass pathogen classifiers, and improving the interpretability of AI-driven clinical decision systems to ensure clinician acceptance (Su et al., 2021). To address these challenges, this study developed and validated a multi-class classification framework based on complete blood count (CBC) for identifying four important pneumonia-causing bacteria (*Pseudomonas aeruginosa* (*P. aeruginosa*), *Escherichia coli* (*E. coli*), *Staphylococcus aureus* (*S. aureus*), and *Streptococcus* pneumoniae (S. pneumoniae)) (Figure 1B). The main contributions include: demonstrating that routine CBC indicators possess discriminatory power to differentiate between the four pathogens, supporting rapid pathogen identification. Designing a two-stage feature selection process to obtain a combination of key biomarkers with low redundancy and high information content, improving model compactness and usability (Mohtasham et al., 2024). Providing global and individual-level interpretability analysis based on SHAP, clarifying the contribution of key features to the prediction of different pathogens, enhancing model transparency and clinical interpretability (Li et al., 2023; Papazoglou and Biskas, 2023).

This study specifically focuses on classifying four WHO-priority Gram-positive and Gram-negative bacteria (*P. aeruginosa*, *E. coli*, *S. aureus*, and S. pneumoniae), which are commonly implicated in hospital-acquired pneumonia. While we recognize that pneumonia can be caused by a broad spectrum of pathogens,the scope of this model is intentionally constrained to a subset of clinically significant bacteria to assess feasibility and performance using readily available CBC data. Furthermore, we acknowledge that not all strains within a species are multidrug-resistant (MDR), and that MDR status can only be definitively confirmed through AST. Thus, although our model does not predict resistance phenotypes directly, it targets organisms frequently associated with MDR in healthcare settings, aiming to support earlier empirical decision-making while reducing unnecessary broad-spectrum antibiotic use. The resulting AI diagnostic model obtained 86.7% overall accuracy in classifying four high-risk bacterial species, surpassing benchmarks set by deep migration learning (Schmidt et al., 2021).

## 2 Materials and methods

### 2.1 Data description and analysis

This study included 1,334 CBC samples from confirmed bacterial pneumonia patients, which included four clinically significant pathogens: *P. aeruginosa*, *E. coli*, *S. aureus*, and S. pneumoniae (Figure 3A). Each record corresponds to a unique patient, and only one CBC measurement per patient was included in the final dataset. Therefore, the independence assumption holds at the patient level, and no patient contributed observations to both the training and test sets. Importantly, all biological specimens for microbiological testing—including sputum or blood cultures—were collected prior to the initiation of any antibiotic treatment. The dataset contains sex and 26 hematological quantitative parameters divided into four functional groups (Table 1): leukocyte parameters (white blood cell count (WBC), absolute neutrophil count (NEUT#), lymphocyte percentage (LYMPH%), absolute monocyte count (MONO#), absolute eosinophil count (EO#), absolute basophil count (BASO#), and their corresponding percentages), erythrocyte indices (red blood cell count (RBC), hemoglobin (HGB), hematocrit (HCT), mean corpuscular volume (MCV), mean corpuscular hemoglobin (MCH), mean corpuscular hemoglobin concentration (MCHC), red cell distribution width–standard deviation (RDW-SD), red cell distribution width–coefficient of variation (RDW-CV)), platelet parameters (platelet count (PLT), mean platelet volume (MPV), platelet–large cell ratio (P-LCR), plateletcrit (PCT), platelet distribution width (PDW)), and immature granulocyte markers (immature granulocyte percentage (IG%), absolute immature granulocyte count (IG#), neutrophil-to-lymphocyte ratio (NLR)).

**TABLE 1:** Demographic and clinical characteristics.

Variable	Sex group		P-value^2^
	Male, N = 697	Female, N = 625	
Age	—	—	0.781
Mean (SD)	68.62 (13.99)	68.40 (14.70)	—
Median (IQR)	70.00 (61.00, 78.00)	72.00 (62.00, 77.00)	—
Leukocyte profile			
WBC	8.27 (6.13, 11.37)	7.39 (5.58, 10.58)	0.229
NEUT#	6.37 (4.19, 9.50)	4.96 (3.13, 8.61)	0.833
LYMPH	1.18 (0.72, 1.66)	1.53 (0.96, 2.08)	0.021
MONO#	0.50 (0.31, 0.71)	0.44 (0.32, 0.61)	0.037
EO#	0.03 (0.00, 0.11)	0.05 (0.01, 0.11)	0.662
BASO#	0.01 (0.00, 0.02)	0.01 (0.00, 0.02)	0.265
NEUT%	77.91 (67.30, 87.11)	68.60 (56.70, 83.10)	<0.001
LYMPH%	14.30 (7.22, 22.10)	21.80 (10.50, 32.64)	<0.001
MONO%	6.40 (4.30, 8.20)	6.10 (4.60, 7.70)	0.557
EO%	0.30 (0.00, 1.40)	0.70 (0.10, 1.70)	0.159
BASO%	0.10 (0.00, 0.20)	0.10 (0.00, 0.20)	0.358
Erythrocyte indices			
RBC	4.47 (3.86, 4.83)	4.27 (3.85, 4.63)	<0.001
HGB	133.00 (114.00, 145.00)	126.00 (112.00, 134.00)	<0.001
HCT	38.80 (33.60, 42.50)	37.20 (33.60, 40.20)	<0.001
MCV	87.10 (84.30, 90.60)	87.20 (83.90, 90.50)	0.724
MCH	29.70 (28.50, 30.70)	29.50 (28.20, 30.50)	0.392
MCHC	339.00 (331.00, 348.00)	336.00 (328.00, 345.00)	0.002
RDW-SD	43.00 (40.00, 47.00)	42.00 (40.00, 45.00)	0.005
RDW-CV	13.00 (13.00, 15.00)	13.00 (13.00, 14.00)	0.003
Platelet parameters			
PLT	205.00 (158.00, 256.00)	239.00 (186.00, 285.00)	<0.001
MPV	9.70 (9.10, 10.30)	9.80 (9.20, 10.50)	<0.001
P-LCR	22.60 (17.40, 27.30)	23.00 (18.60, 28.80)	0.017
PCT	0.20 (0.16, 0.24)	0.23 (0.18, 0.28)	<0.001
PDW	10.60 (9.40, 11.80)	10.80 (9.80, 12.20)	0.135
IG%	0.30 (0.20, 0.50)	0.30 (0.20, 0.50)	0.186
IG#	0.03 (0.02, 0.05)	0.02 (0.01, 0.05)	0.384
NLR	5.42 (3.03, 12.05)	3.15 (1.76, 7.86)	<0.001

Data quality control was strictly enforced; samples with missing values (5–23 missing entries per feature) were excluded. To address class imbalance in the target variables, stratified sampling was used during modeling to reduce bias (Aguiar et al., 2023). A hybrid feature selection technique improved model performance: LASSO regression removed collinear variables (Bainter et al., 2023), and the Boruta algorithm discovered nonlinear correlations (Fahimifar et al., 2022), yielding a consensus feature subset.

Histograms show right-skewed distributions for many parameters (e.g., HGB and MCV), showing a concentration of values in lower ranges and outliers at higher extremes, signifying data skewness or potential anomalies. These findings necessitate further standardization or transformation to improve classifier performance (Azad et al., 2021). Collectively, these preliminary analyses provide important recommendations for improving pathogen categorization systems.

This dataset provides a robust informative basis for predicting four different bacterial infections via regular blood measurements. The scientific rationale for selecting this dataset is based on the strong clinical utility of hematological data for rapid diagnostic frameworks (Khalid et al., 2021), which can aid in clinical decision-making, improve therapeutic efficiency through timely pathogen-specific interventions, and significantly reduce misdiagnosis risks in resource-constrained healthcare settings.

In all experiments, we followed a leakage-free workflow. We first split the data, then applied preprocessing (e.g., scaling) and feature selection using the training set only. Hyperparameters were optimized via cross-validation (CV) on the training set. Finally, the selected model was refit on the full training set and evaluated on the held-out test set. At no stage were the test data used for preprocessing, feature selection, or model tuning.

### 2.2 Feature selection

This work used a dual-strategy method for high-robustness feature screening, combining the Boruta all-relevant feature selection algorithm with LASSO regression.

The Boruta algorithm (Zhou et al., 2023), which uses 100 iterations of shadow feature permutation tests, identifies key variables from the entire dataset, including core inflammatory indicators (white blood cell count, neutrophil absolute count and percentage) and immune cell parameters (lymphocyte and monocyte absolute counts and percentages) (Figure 3B).

Prior to LASSO-based feature selection, all continuous hematological variables were standardized using z-score normalization. Importantly, the mean and standard deviation were estimated using the training data only, and the same transformation was then applied to the test data to avoid information leakage.

LASSO regression with cross-validation (LASSOCV) was used to penalize collinear and low-contribution variables via L1 regularization (Wang et al., 2024). The optimization path automatically retains only high-impact predictors. This phase further highlighted erythrocyte system parameters (HGB and HCT) and inflammatory dynamics indicators (NEUT# and MONO#).

These two techniques use complementary dimensions to select features: Boruta values biological relevance, whereas LASSO emphasizes prediction efficiency. A Venn analysis extracted the intersection of features identified by both methods, yielding consensus variables, as shown in Table 2 (Barbieri et al., 2024). This dual-validation mechanism overcomes single-algorithm selection bias, establishing a high-reliability feature benchmark for infectious pathogen classification.

**TABLE 2:** Feature selection.

Variable	*P. aeruginosa*	*E. coli*	*S. aureus*	*S. pneumoniae*	P-value^2^
WBC	8.64 (6.12, 11.48)	7.59 (5.84, 10.40)	8.69 (7.04, 12.88)	8.24 (5.54, 11.37)	0.009
NEUT#	6.56 (4.29, 9.72)	5.21 (3.34, 8.57)	6.86 (4.58, 10.28)	6.29 (3.52, 9.46)	0.895
MONO#	0.49 (0.30, 0.66)	0.45 (0.33, 0.62)	0.56 (0.33, 0.77)	0.45 (0.30, 0.66)	<0.001
LYMPH%	13.84 (8.82, 22.44)	19.32 (9.31, 31.48)	14.90 (8.60, 23.80)	15.00 (7.22, 24.84)	<0.001
MONO%	6.60 (4.10, 8.30)	6.20 (4.70, 7.80)	6.00 (4.90, 8.80)	6.40 (3.90, 8.10)	0.869
BASO%	0.07 (0.00, 0.20)	0.10 (0.00, 0.20)	0.14 (0.10, 0.30)	0.10 (0.00, 0.20)	0.179
RBC	4.08 (3.46, 4.69)	4.32 (3.90, 4.72)	4.43 (3.91, 4.85)	4.34 (3.92, 4.79)	0.015
HGB	120 (95.50, 137.00)	128 (115.00, 139.00)	131 (114.00, 146.00)	130 (116.00, 143.00)	<0.001
HCT	35.60 (29.38, 40.80)	37.90 (34.10, 41.00)	39.50 (34.10, 42.10)	37.60 (34.30, 42.00)	0.008
MCV	87.00 (83.30, 90.58)	87.15 (84.03, 90.30)	87.40 (84.00, 91.90)	87.10 (84.60, 90.60)	0.360
MCH	29.20 (27.80, 30.10)	29.60 (28.43, 30.60)	29.80 (28.30, 30.80)	29.80 (28.70, 30.70)	0.385
MCHC	333 (323.00, 342.00)	338 (330.00, 346.00)	338 (329.00, 348.00)	339 (331.00, 348.00)	<0.001
RDW-SD	45.00 (41.25, 49.00)	43.00 (40.00, 45.00)	41.00 (39.00, 46.00)	42.00 (40.00, 46.00)	<0.001
P-LCR	22.00 (17.63, 26.85)	22.40 (18.00, 27.30)	23.40 (18.70, 27.20)	23.70 (18.40, 28.90)	0.113
PCT	0.22 (0.17, 0.29)	0.22 (0.18, 0.27)	0.23 (0.17, 0.26)	0.21 (0.15, 0.25)	0.003
PDW	10.70 (9.13, 11.70)	10.60 (9.60, 11.90)	10.90 (9.90, 12.00)	10.70 (9.60, 12.30)	0.516
NLR	5.71 (3.00, 9.44)	3.62 (1.89, 8.85)	5.10 (2.82, 9.39)	5.10 (2.55, 12.20)	0.428

### 2.3 Model development: ensemble framework with metaheuristic optimization and interpretable AI

This study proposes a hybrid machine learning framework (PreBP) that integrates metaheuristic optimization (Rezk et al., 2024) and stacked ensemble learning (Goliatt et al., 2023) for multiclass pathogen classification. The goal was to differentiate among *P. aeruginosa*, *E. coli*, *S. aureus*, and S. pneumoniae infections using CBC-derived immune signatures.

#### 2.3.1 Base learner selection and optimization

Four candidate machine learning algorithms were chosen as base learners based on their ability to handle structured clinical data and capture complex non-linear correlations: extremely randomized trees (ET), extreme gradient boosting (XGBoost) (Hakkal and Lahcen, 2024), random forest (RF), and adaptive boosting (AdaBoost) (Han et al., 2022).

**FIGURE 2:**
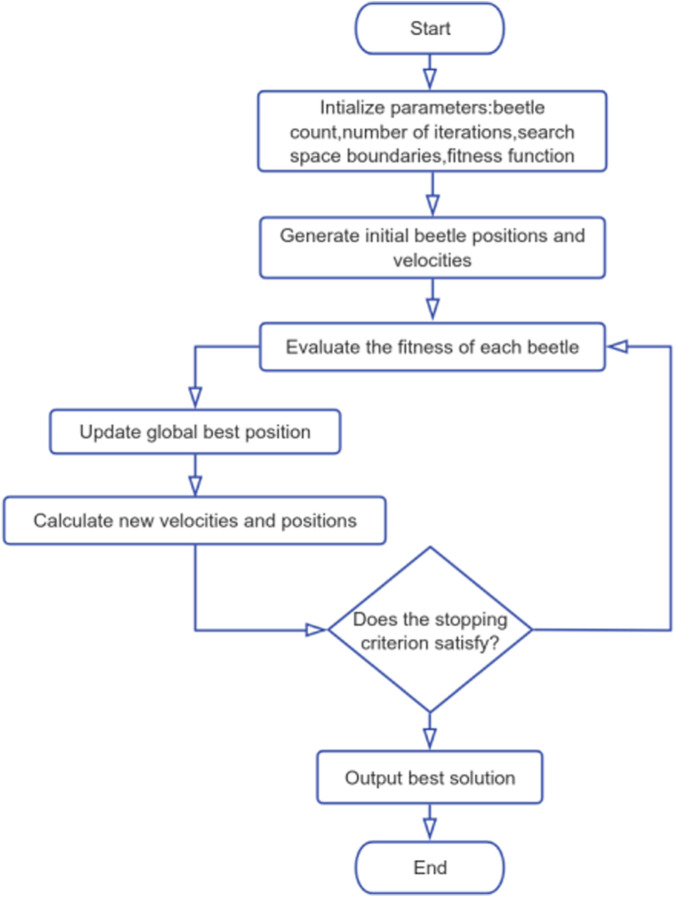
DBO Algorithm Workflow. The Dung Beetle Optimizer (DBO) algorithm mimics the behavioral patterns of dung beetles, employing rolling dynamics (directional movement with stochastic perturbations), a dance-inspired operator to balance exploration and exploitation, breeding-phase local optimization, and resetting low-fitness agents to enhance global search capabilities. This dynamic optimization framework enables robust problem-solving and is particularly effective for high-dimensional machine learning tasks.

To improve prediction stability, we used a two-stage optimization approach that included five-fold stratified cross-validation and the Dung Beetle Optimizer (DBO). We used five-fold stratified cross-validation on the training set for hyperparameter tuning and model selection. After tuning, the selected models were refit on the full training set, and the final performance was evaluated once on the predefined held-out test set. The DBO algorithm (Figure 2) optimizes hyperparameters by dynamically balancing global search capabilities with localized refinement (Xue and Shen, 2022). In the initialization phase, the algorithm randomly generates the position and velocity vectors of the dung beetle population. By introducing random perturbation factors, elite retention strategies, and mechanisms based on iterative fitness evaluation (such as cross-validation F1 scores), the algorithm continuously updates the search trajectory until convergence. Following DBO optimization, the top three base learners—ET, XGBoost, and AdaBoost—were selected for inclusion in the ensemble based on cross-validation performance. For a fair comparison, all baseline models were trained and evaluated under the same data split and the same five-fold stratified cross-validation scheme. All cross-validation procedures were conducted within the training set only for hyperparameter tuning and model selection. The held-out test set was used once for final performance reporting. Hyperparameters for both PreBP and baselines were optimized using the same DBO procedure, with an identical optimization budget and objective metric.

#### 2.3.2 Stacked ensemble construction

**FIGURE 3:**
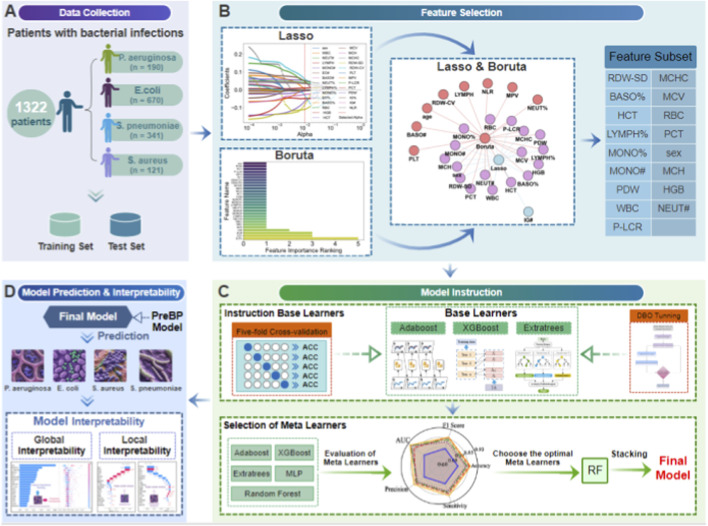
An interpretable AI pipeline for bacterial infection diagnosis that integrates optimized feature selection and stacked ensemble learning **(A)** Data collection. A total of 1,332 patients (123 with *Pseudomonas aeruginosa*, 234 with *Escherichia coli*, 122 with *Staphylococcus aureus*, and 89 with *Streptococcus pneumoniae*) were included and split into training/test sets. **(B)** Feature selection. LASSO regression and the Boruta algorithm were used to identify consensus variables via a Venn diagram. **(C)** Model instruction. DBO-optimized base learners fused with 5-fold cross-validation; meta-learner selected via radar plot evaluation. **(D)** Model prediction & interpretation: PreBP predicts bacterial infections with global SHAP and local instance-level explanations.

To improve diagnostic robustness, we designed a two-phase stacked ensemble architecture (Figure 3C). In the first step, the outputs (probabilistic class predictions) of the selected base learners were used as inputs for the meta-learner. To prevent information leakage in stacking, we trained the meta-learner on out-of-fold (OOF) class probabilities generated by five-fold stratified cross-validation on the training set. In each fold, base learners were fit on the remaining K−1 folds and used to predict the held-out fold; aggregated OOF probabilities for all training samples were then used as meta-features. At inference, base learners were refit on the full training set with optimized hyperparameters to generate test-set probabilities, which were passed to the meta-learner for final predictions. The second step involved a systematic comparative evaluation of five prospective meta-learners (DBO-XGBoost, DBO-ET, DBO-RF, DBO-AdaBoost, and DBO-MLPNN), which culminated in the integration of the best-performing meta-learner. Experimental results show that the DBO-optimized random forest (DBO-RF) meta-learner has better generalization ability. DBO-RF is the most effective meta-learner with a macro F1 score of 0.86, surpassing all individual base models. This hierarchical integration technique provides persistence and generalization capabilities across multiple pathogen types.

## 3 Results

### 3.1 Predictive model evaluation

Using the predefined data split, PreBP achieved an accuracy of 86.7%, precision of 87.1%, recall of 86.7%, F1 of 86.0%, and a macro-OVR AUC of 0.920 for four-class pathogen classification. Across the same evaluation protocol, PreBP outperformed ET, XGBoost, RF, AdaBoost, and MLPNN. Leveraging routinely measured CBC variables, PreBP can deliver timely and interpretable predictions once CBC results become available. SHAP-based explanations (Ponce-Bobadilla et al., 2024) reveal both cohort-level discriminative patterns and patient-specific drivers (Figure 3D), offering complementary evidence alongside culture-based testing. This study proposes and validates a modeling framework based on routine blood test indicators, aiming to address the limitations of traditional pathogen identification methods in terms of timeliness and accessibility (Shojaei et al., 2023).

### 3.2 Superior diagnostic performance of DBO-Stacking model

**FIGURE 4:**
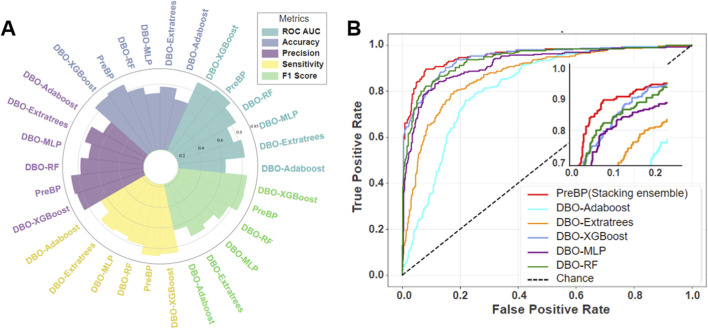
Comparative analysis of DBO-optimized models and the PreBP ensemble: ROC evaluation and diagnostic metrics **(A)** The figure compares evaluation metrics (ROC AUC, accuracy, precision, sensitivity, and F1 score) of five DBO-optimized multipathogen prediction models (AdaBoost, Extratrees, MLP, RF, and XGBoost) and a novel ensemble model (PreBP), demonstrating the superior diagnostic performance of PreBP, which validates its efficacy in pathogen prediction. **(B)** ROC curves of five baseline models and the newly developed PreBP ensemble model, with PreBP achieving the highest AUC, confirming its optimal discriminative capacity.

Using a clinical dataset (n = 1,334), we implemented a stratified sampling strategy to partition the data into training and independent test sets, ensuring a consistent distribution of multiple pathogens. We systematically evaluated multiclass diagnostic performance by comparing five DBO-optimized baseline models (DBO-XGBoost, DBO-RF, DBO-ExtraTrees, DBO-MLPNN, DBO-AdaBoost) and a DBO-optimized stacked ensemble model (DBO-Stacking) (Table 3; Figure 4).

**TABLE 3:** Evaluation metrics for the six models.

ML algorithms	Accuracy	ROC-AUC	Sensitivity	Precision	F1 score
DBO-AdaBoost	0.666	0.637	0.666	0.640	0.636
DBO-extratrees	0.773	0.828	0.773	0.769	0.743
DBO-XGBoost	0.850	0.894	0.850	0.850	0.849
DBO-MLP	0.710	0.748	0.748	0.701	0.701
DBO-RF	0.783	0.851	0.783	0.775	0.771
PreBP	**0.867**	**0.920**	**0.867**	**0.871**	**0.860**

Bold values indicate the best performance (highest value) among the compared models for each evaluation metric.

As shown in Table 3, the DBO-Stacking model demonstrated significant diagnostic advantages in the independent test set. The model achieved an AUC of 0.920, outperforming the best-performing baseline model in our comparative experiments (DBO-XGBoost: AUC = 0.894; Δ = 0.026). The model also obtained an accuracy of 86.7% and a precision of 87.1%, with a macro-F1 score of 0.860, indicating strong overall multiclass discrimination in this dataset. These results suggest that the proposed DBO-Stacking framework can provide reliable probabilistic predictions once CBC results are available and may provide supplementary information to support pathogen-oriented decision-making within the scope of the current evaluation setting.

### 3.3 Enhanced sensitivity and ROC analysis of ensemble learning

As shown in Figure 4A, DBO-Stacking showed outstanding sensitivity (0.867) and false-negative rates (FNR = 13.3%), with improvements of 9.4% and 8.4% above DBO-ExtraTrees (sensitivity = 0.773) and DBO-RF (sensitivity = 0.783), respectively. Figure 4B reveals that its ROC curve is closest to the upper-left quadrant, maintaining true-positive rates (TPRs) > 0.85 even at high false-positive rates (FPRs>0.8), whereas baseline models (e.g., DBO-ExtraTrees) show TPR deterioration in this region. These findings show that the ensemble framework, which uses dynamic host immune parameters (e.g., the NLR and PDW), effectively mitigates the synergistic risks of missing and misdiagnosis while offering high-robustness decision support for early precision intervention in resistant infections.

### 3.4 Global SHAP interpretation reveals pathogen-specific feature attribution patterns and sex-stratified differences

SHAP analysis was performed for the final output of PreBP (i.e., the stacked ensemble’s meta-learner output). When computing SHAP values, we treated the fitted base learners and the trained meta-learner as a single composite prediction function that maps CBC features to class probabilities; therefore, SHAP values are reported with respect to the original CBC input features, reflecting their contributions to the final ensemble predictions.

**FIGURE 5:**
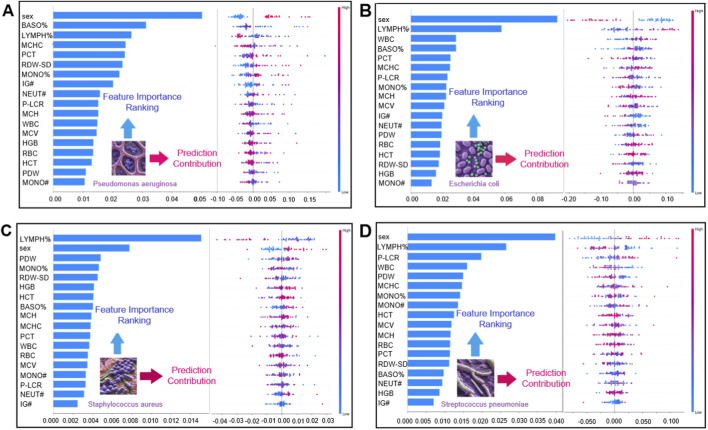
Global feature importance and impact direction analysis in bacterial infection prediction. Panel Merged bar chart and honeycomb plot with a shared y-axis for straightforward interpretability **(A)** Global feature interpretation for *P. aeruginosa* infection prediction. The left bar plot displays feature importance rankings, whereas the right hive plot visualizes the impact direction (positive/negative) and value-dependent prediction trends. **(B)** Global feature interpretation for *E. coli* infection prediction. The left bar plot highlights feature importance rankings, with the right hive plot mapping impact direction and value-influence trends. **(C)** For *S. aureus* infection prediction, the left bar plot ranks feature importance, whereas the right hive plot shows impact direction (positive/negative) and value-dependent prediction trends. **(D)** For *S. pneumoniae*, the left bar plot similarly ranks key features, with the right hive plot mapping directional impacts and feature-value influences.

SHAP analysis (Figure 5) identified class-specific patterns of influential CBC variables for discriminating the four bacterial pneumonia pathogen categories in this study. BASO% and RDW-SD ranked among the most influential features (by mean absolute SHAP value) for the *P. aeruginosa* class, whereas LYMPH% and WBC were among the leading contributors for the *E. coli* class. For *S. aureus*, LYMPH% and PDW showed prominent contributions, and for S. pneumoniae, LYMPH% and P-LCR were among the more influential features. These SHAP values provide *post hoc*, model-based interpretations of how CBC variables contribute to the model’s predicted class probabilities.

We further examined sex-stratified patterns in this study. The distribution of pathogen labels differed between males and females, with *P. aeruginosa* cases relatively more frequent among males and *E. coli* and *S. aureus* cases relatively more frequent among females in this dataset. This observation is descriptive and may reflect confounding factors (e.g., age, comorbidities, exposure history, or sampling practices) rather than sex-specific biological susceptibility. In the SHAP analysis, PDW showed notable contributions to *S. aureus* class probabilities, and WBC-related variables also contributed to the discrimination of pathogen classes; however, these associations reflect model attributions rather than mechanistic pathways.

### 3.5 Individualized SHAP profiling illustrates patient-level feature attributions

**FIGURE 6:**
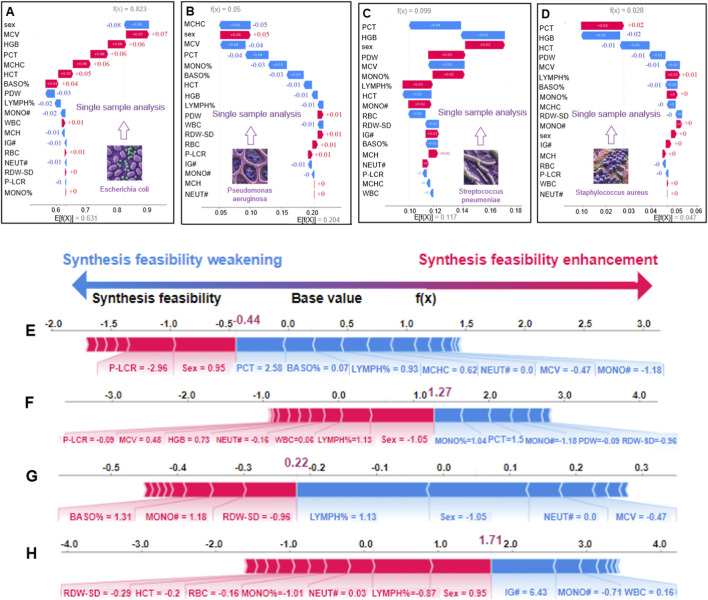
Local feature contribution analysis across bacterial infections **(A)** Panel A shows the local instance-level interpretation for an *E. coli*-infected patient’s blood sample. **(B)** Panel B illustrates the local interpretation for a *P. aeruginosa*-infected sample, mapping feature-specific impacts on prediction outcomes. **(C)** Panel C presents single-sample analysis for *S. pneumoniae* infection. **(D)** Panel D shows an instance-level explanation for an *S. aureus*-infected patient. **(E)** The figure analyzes a single blood sample from a *P. aeruginosa*-infected patient. **(F)** The figure highlights feature-specific impacts (direction and magnitude) for an *E. coli*-infected patient’s blood sample. **(G)** For an *S. aureus*-infected patient, the visualization maps critical features and their contribution polarity (positive/negative) to the diagnostic outcome. **(H)** Analysis of an *S. pneumoniae*-infected sample reveals dominant features and their predictive influence directions.

We used SHAP waterfall plots to illustrate feature attributions for individual predictions. For an *E. coli* case (Figure 6A), MCV had the largest positive SHAP contribution (+0.07) to the predicted probability of the *E. coli* class, whereas PDW (−0.03) and LYMPH% (−0.02) showed negative contributions. Platelet-related parameters (PCT, +0.06; MCHC, +0.06) also contributed positively to the same class probability. For an S. pneumoniae case (Figure 6C), PDW (+0.03) contributed positively, whereas PCT (−0.04) contributed negatively. These examples illustrate how routine CBC variables combine to influence the model’s class probability outputs at the individual-sample level (Figures 6A–D).

Force plots (Figures 6E–H) further visualize patient-level attributions for representative cases. In a *P. aeruginosa* case (Figure 6E), P-LCR showed a positive SHAP contribution to the predicted probability of the *P. aeruginosa* class, whereas PCT, BASO%, and LYMPH% contributed negatively. For an *S. aureus* case (Figure 6G), RDW-SD showed a positive contribution, whereas LYMPH% contributed negatively. Overall, the global and case-level SHAP results improve the transparency of PreBP by linking predictions to CBC inputs. These attributions are model-based associations and should be interpreted as hypothesis-generating; additional clinical or experimental validation is required before drawing mechanistic or causal conclusions.

### 3.6 Optimized ensemble learning framework for enhanced diagnostic generalization

**FIGURE 7:**
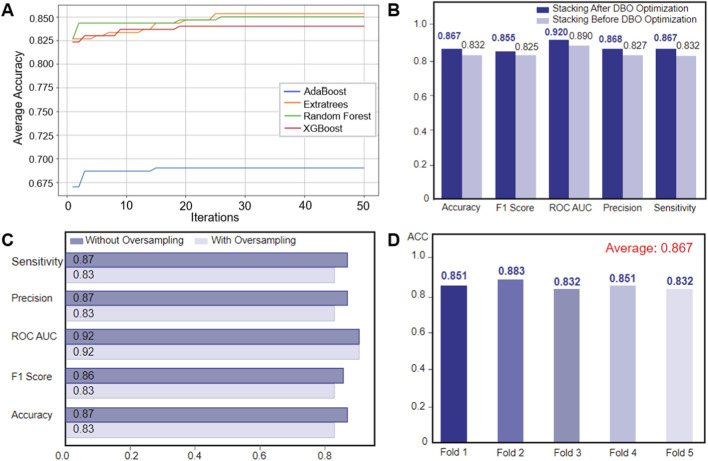
Model optimization and validation workflow **(A)** Convergence trends of classifier performance. The figure illustrates the dynamic changes in the average classification accuracy of four DBO-optimized base classifiers (AdaBoost, ExtraTrees, Random Forest, XGBoost) across iterations. **(B)** Performance enhancement of the stacking model post-DBO optimization. Bar charts compare key metrics of the stacking ensemble model before and after DBO optimization: accuracy (+3.5%), F1 score (+3%), ROC AUC (+3%), precision (+4.1%), and sensitivity (+3.5%). **(C)** Impact of oversampling on classification performance. **(D)** Cross-validation stability and generalization capability.

The experimental framework combines the Dung beetle optimizer algorithm for hyperparameter optimization in four machine learning architectures: ExtraTrees, XGBoost, random forest, and AdaBoost. The parameter search boundaries are referenced in Table 2, and the optimization performance is evaluated by repeating fivefold cross-validation. The iteration termination criterion is set to 50 cycles. As shown in the convergence curve of Figure 7A, all classifiers converge to a stable accuracy after the 27th iteration cycle. Among them, AdaBoost shows fast early optimization, whereas ExtraTrees shows gradual but continuous improvement and finally achieves excellent generalization ability on the reserved dataset. The key parameter adjustments that occurred in this process include the number of weak classifiers of AdaBoost, the maximum depth of ET, and the learning rate of XGBoost (the specific configurations can be found in Table 4). Optimized benchmark testing shows that all classifiers achieve an accuracy improvement of 3.2%–3.8% compared with their default configurations, validating the effectiveness of DBO in efficient parameter searching. The subsequent ensemble architecture integrates these optimized models via stacked generalization, which results in strong predictive synergy—achieving an area under the curve (AUC) of 0.917–0.923 while maintaining F1 score stability in the range of 0.853–0.867.

**TABLE 4:** Optimizing the hyperparameters of the four base learners via the DBO algorithm.

ML algorithms	Hyperparameters	Scope of values	Hyperparameters
AdaBoost	n_estimators	(50, 500)	173.557
	learning_rate	(0.01, 1.0)	0.214
Extratrees	n_estimators	(100, 500)	196.959
	max_depth	(3, 15)	13.025
	min_samples_split	(2, 20)	15.815
	min_samples_leaf	(1, 15)	12.841
Xgboost	n_estimators	(100, 500)	195.352
	max_depth	(3, 15)	7.479
	learning_rate	(0.01, 1.0)	0.216
	Subsample	(0.6, 1.0)	0.641
	colsample_bytree	(0.6, 1.0)	0.837
RF	n_estimators	(100, 500)	352.799
	max_depth	(3, 15)	13.876
	min_samples_split	(2, 20)	3.980
	min_samples_leaf	(1, 15)	1.965

## 4 Discussion

### 4.1 DBO-driven hyperparameter optimization enhances PreBP model performance

This paper offers an advanced hybrid stacking ensemble model that combines numerous base learners and meta-learners and optimizes them using the DBO algorithm to considerably increase model performance. To assess the actual impact of DBO optimization, we systematically analyze the models’ performance before and after optimization (Figure 7B). The results show that the accuracy of the model after DBO optimization is improved by 4.1%, the sensitivity and accuracy are improved by 3.5%, and the AUC and F1 scores are improved by 3%, which shows that DBO has a strong advantage in hyperparameter optimization and can effectively find the best configuration to enhance model performance. To ensure a fair comparison, all baseline models were tuned using the same five-fold stratified cross-validation scheme and the same DBO-based hyperparameter optimization procedure (with a matched optimization budget and objective metric) as PreBP.

The core contribution of this study is the proposal of an innovative ensemble optimization strategy to address complex machine learning tasks. Traditional hyperparameter optimization methods usually have difficulty efficiently searching a large parameter space, which affects the final model performance. By introducing DBO, this study not only significantly improves the predictive ability of the stacking ensemble model but also provides a solid theoretical foundation for future hyperparameter optimization. Through refined parameter adjustment, DBO fully releases the potential of machine learning models and provides new technical support for intelligent decision-making in various industries.

### 4.2 SMOTE amplifies noise in bacterial infection classification

In clinical machine learning applications, class imbalance poses a significant threat to the effectiveness of predictive models. To address the imbalance problem in a bacterial infection dataset, we applied the synthetic minority oversampling technique (SMOTE), which generates synthetic minority samples through feature space interpolation.

However, the experimental results (Figure 7C) show that the performance of the stacked ensemble model trained on the SMOTE balanced dataset decreases in terms of all the metrics: the average accuracy and precision decrease by 4%, whereas the macro F1 score decreases by 3%. This demonstrates that, while SMOTE reduces class imbalance by increasing minority sample sizes, it may also introduce noise or distort the original data distribution, increasing the risk of overfitting and decreasing generalizability. Furthermore, SMOTE’s local interpolation method ignores the global data structure, thereby exacerbating minority learning bias.

### 4.3 Fivefold cross-validation confirms ensemble robustness for pathogen prediction

To further examine the robustness of the proposed ensemble, we performed five-fold stratified cross-validation within the training set during model development. The experimental findings (Figure 7D) revealed that the ensemble was highly consistent, with an interfold accuracy variance of only 2.1% (0.832–0.883) and an average accuracy of 0.867. These measures illustrate the model’s resilience in multiclass pathogen prediction as well as its adaptability to changing data. Compared with single-model techniques, the hybrid stacking ensemble strategy successfully synergizes the benefits of base learners, increasing classification accuracy and flexibility to data changes. These results indicate that the ensemble performance is stable across cross-validation folds. Final performance metrics were reported based on the predefined held-out test set.

## 5 Conclusion

This study proposes PreBP, a pneumonia pathogen classification model based on feature selection and metaheuristic optimization, which enhances multiclass bacterial infection prediction through a hybrid stacked ensemble (ExtraTrees/RF/MLPNN/XGBoost) integrated with the Dung Beetle Optimizer (DBO) and five-fold cross-validation. Experimental results reveal that PreBP outperforms single learners on the independent test set, proving it as a trustworthy tool for quick pathogen detection. SHAP-based interpretability analyses were used to summarize model-level feature attributions associated with key CBC parameters to the predicted probabilities of each pathogen class, thereby improving the transparency of model outputs and facilitating clinical interpretation.

The main contributions of this work are threefold. We develop and evaluate a DBO-tuned training pipeline for hyperparameter selection in the proposed multiclass prediction setting. We present an interpretable stacked ensemble framework for CBC-based four-class pathogen identification under the evaluation protocol used in this study. We also report both class-wise global feature importance rankings and case-level explanations using SHAP to illustrate how CBC variables contribute to model predictions. This study focuses on four target pathogens in the current dataset; future work should expand pathogen coverage, include external and prospective validation, and explore performance on resistant strains when appropriate reference labels are available.

## Data Availability

The raw data supporting the conclusions of this article will be made available by the authors, without undue reservation.

## Glossary

WHO: World Health Organization
CBC: complete blood count
AMR: antimicrobial resistance
*P. Aeruginosa*: *Pseudomonas aeruginosa*
*E. Coli*: *Escherichia coli*
*S. Aureus*: *Staphylococcus aureus*
*S. Pneumoniae*: *Streptococcus pneumoniae*
PDW: platelet distribution width
NLR: neutrophil-to-lymphocyte ratio
DBO: Dung Beetle Optimizer
HAIs: hospital-acquired infections
AST: antimicrobial susceptibility testing
PCT: procalcitonin
ML: Machine learning
MDROs: multidrug-resistant organisms
PDW: platelet distribution width
HIF: Host Immune Fingerprint
SHAP: SHapley Additive exPlanations
LIME: Local Interpretable Model-agnostic Explanations
HGB: hemoglobin
HCT: hematocrit
ET: Extremely Randomized Trees
XGBoost: Extreme Gradient Boosting
RF: Random Forest
AdaBoost: Adaptive Boosting
BASO%: basophil percentage
LYMPH%: lymphocyte percentage
WBC: white blood cell count
PDW: platelet distribution width
P-LCR: platelet-large cell ratio
RDW: red cell distribution width
MCV: mean corpuscular volume
RDW-SD: red cell distribution width standard deviation
NEUT%: Neutrophil percentage
MCHC: mean corpuscular hemoglobin concentration
MONO#: monocyte counts
SMOTE: Synthetic Minority Oversampling Technique
CV: cross-validation
